# Motion-Induced Errors in Buoy-Based Wind Measurements: Mechanisms, Compensation Methods, and Future Perspectives for Offshore Applications

**DOI:** 10.3390/s26030920

**Published:** 2026-01-31

**Authors:** Dandan Cao, Sijian Wang, Guansuo Wang

**Affiliations:** 1 East China Sea Forecasting and Disaster Reduction Center, Ministry of Natural Resources, No. 1593 Haigang Avenue, Pudong New District, Shanghai 201306, China; 2 Observation and Research Station of Huaniaoshan East China Sea Ocean-Atmosphere Integrated Ecosystem, Ministry of Natural Resources, Shanghai 201306, China; 3 Key Laboratory of Marine Ecological Monitoring and Restoration Technologies, Ministry of Natural Resources, Shanghai 201206, China

**Keywords:** buoy wind measurement, motion-induced error, six-degree-of-freedom motion, attitude compensation, floating LiDAR, turbulence intensity, offshore wind energy

## Abstract

Accurate measurement of sea-surface winds is critical for climate science, physical oceanography, and the rapidly expanding offshore wind energy sector. Buoy-based platforms—moored meteorological buoys, drifters, and floating LiDAR systems (FLS)—provide practical alternatives to fixed offshore structures, especially in deep water where bottom-founded installations are economically prohibitive. Yet these floating platforms are subject to continuous pitch, roll, heave, and yaw motions forced by wind, waves, and currents. Such six-degree-of-freedom dynamics introduce multiple error pathways into the measured wind signal. This paper synthesizes the current understanding of motion-induced measurement errors and the techniques developed to compensate for them. We identify four principal error mechanisms: (1) geometric biases caused by sensor tilt, which can underestimate horizontal wind speed by 0.4–3.4% depending on inclination angle; (2) contamination of the measured signal by platform translational and rotational velocities; (3) artificial inflation of turbulence intensity by 15–50% due to spectral overlap between wave-frequency buoy motions and atmospheric turbulence; and (4) beam misalignment and range-gate distortion specific to scanning LiDAR systems. Compensation strategies have progressed through four recognizable stages: fundamental coordinate-transformation and velocity-subtraction algorithms developed in the 1990s; Kalman-filter-based multi-sensor fusion emerging in the 2000s; Response Amplitude Operator modeling tailored to FLS platforms in the 2010s; and data-driven machine-learning approaches under active development today. Despite this progress, key challenges persist. Sensor reliability degrades under extreme sea states precisely when accurate data are most needed. The coupling between high-frequency platform vibrations and turbulence remains poorly characterized. No unified validation framework or benchmark dataset yet exists to compare methods across platforms and environments. We conclude by outlining research priorities: end-to-end deep-learning architectures for nonlinear error correction, adaptive algorithms capable of all-sea-state operation, standardized evaluation protocols with open datasets, and tighter integration of intelligent software with next-generation low-power sensors and actively stabilized platforms.

## 1. Introduction

With growing attention to climate change and the accelerating shift toward renewable energy, accurate sea-surface wind measurements have become essential for oceanographic research and offshore engineering applications [1]. Sea-surface winds drive key physical processes, like air–sea interaction, wind-generated waves, and ocean circulation [2], and play a decisive role in wind resource assessment, marine infrastructure safety, extreme-weather prediction, and secure maritime navigation [3,4]. The offshore wind sector, in particular, is expanding rapidly from coastal waters into deeper ocean regions, creating an urgent need for reliable wind observations in areas far from shore [5,6,7]. Traditional fixed platforms, such as meteorological masts, however, become prohibitively expensive in deep water, and their deployment options are limited by seabed conditions and permitting constraints [8]. These limitations have motivated the development of mobile observing systems that can acquire high-quality wind data over extended periods in previously inaccessible locations [9].

Buoy-based platforms now form a vital component of the Global Ocean Observing System (GOOS). Moored meteorological buoys, drifting buoys, and the increasingly popular floating LiDAR systems (FLS) offer flexible deployment, relatively low capital costs, and the ability to operate where fixed structures cannot [10,11]. These advantages come with a fundamental trade-off. Floating on an energetic and continuously evolving sea surface, every buoy experiences pronounced pitch, roll, heave, surge, sway, and yaw—a six-degree-of-freedom (6-DOF) dynamics driven by the combined forcing of wind, waves, and currents [12]. Such motion directly interferes with the sensors mounted on the platform, introducing systematic errors that currently represent a major bottleneck for buoy-based wind observation [13,14]. While mechanical stabilization systems (e.g., gimbals) can theoretically reduce sensor tilt, they are not widely adopted in operational buoy networks [15]. This is primarily due to challenges in maintaining long-term mechanical robustness in harsh marine environments and the difficulty of stabilizing against multi-axis, broadband wave-driven motion [16]. Furthermore, for modern technologies such as scanning LiDAR, measurement errors are governed not only by sensor-body tilt but also by sampling-geometry effects [17], meaning that physical leveling alone is insufficient to eliminate motion-induced distortions. Understanding the origin of these errors and developing effective compensation methods have therefore become pressing scientific and engineering priorities.

How exactly does platform motion degrade the measured wind signal? The answer involves multiple, interacting physical pathways. When the buoy tilts in pitch or roll, the coordinate frame of the anemometer rotates with it, causing the horizontal wind vector to be projected incorrectly and producing a systematic low bias [18,19]. Superimposed on this geometric effect is a kinematic one: the buoy’s translational and rotational velocities add spurious components to the relative wind speed sensed by the instrument, an effect that becomes especially significant under light-wind conditions [20,21]. More subtly, buoy motions introduce artificial velocity fluctuations within the frequency range relevant to atmospheric turbulence analysis. Although structural vibrations may occur at higher frequencies, the motion components that contaminate wind measurements are primarily those overlapping with the turbulence-relevant band, typically on the order of 0.1–1 Hz. These motion-induced fluctuations fall within the same spectral range used to estimate turbulence statistics, thereby inflating derived quantities such as turbulence intensity (TI) by approximately 15–50% [9,17,22,23]. For scanning remote-sensing instruments like LiDAR, the problem is compounded: platform motion misaligns the laser beams and distorts the sampling geometry, leading to errors in reconstructed wind profiles that are qualitatively different from those affecting point sensors [5,17,24].

The impact of these errors extends beyond scientific uncertainty to real economic and operational risk. Since wind speed is fundamental for resource evaluation and Annual Energy Production (AEP) estimation, even a 1% measurement bias may produce a 2–3% deviation in AEP calculations [25,26]. At the scale of large offshore wind farms, this corresponds to substantial financial consequences. Overestimation of turbulence intensity further affects fatigue-load assessments and turbine selection, increasing operational costs and long-term risks [9,22,27]. Beyond offshore wind applications, accurate buoy-based wind measurements are essential for enhancing integrated marine observing networks, improving early-warning systems for marine hazards, and advancing scientific understanding of air–sea interaction processes [10,28]. In each of these domains, uncompensated motion errors translate directly into degraded products and increased uncertainty.

Recognizing these challenges, researchers have devoted considerable effort over the past several decades to characterizing motion-induced errors and devising correction strategies. Yet despite notable advances, significant gaps remain. Our understanding of how different error mechanisms interact across varying sea states, sensor types, and platform designs is still incomplete. Published compensation techniques span a wide range—from simple geometric transformations to sophisticated machine-learning algorithms—but direct, standardized comparisons are rare. This paper, therefore, aims to provide a unified review of the current state of knowledge, synthesizing the physical mechanisms of motion-induced error, the evolution and performance of compensation methods, and the outstanding challenges that must be addressed before buoy-based wind measurements can achieve operational-grade accuracy and reliability.

The remainder of the paper is organized as follows. Section 2 describes the scope of the review and the literature sources consulted. Section 3 introduces buoy-based wind-measurement systems and their characteristic motion behavior in the marine environment. Section 4 presents a mechanistic classification of attitude-induced measurement errors. Section 5 reviews compensation techniques, tracing their development from foundational physical-model approaches through modern data-driven methods. Section 6 identifies the major challenges that remain unsolved, and Section 7 outlines priority directions for future research. Section 8 concludes with a summary of key findings and their implications.

## 2. Scope and Literature Sources

This review offers a comprehensive synthesis of research concerning motion-induced uncertainties in buoy-based wind measurements and the corresponding error-compensation methodologies. The analysis focuses on three primary dimensions: (i) the physical pathways through which buoy dynamics introduce measurement bias and signal distortion, (ii) the development, classification, and comparative performance of existing correction techniques, and (iii) unresolved scientific and engineering issues that constrain routine operational implementation. The relevant literature was selected through a comprehensive survey of academic databases, including Web of Science, Scopus, and IEEE Xplore, complemented by backward citation tracing of seminal contributions and examination of technical reports issued by industrial bodies (e.g., Carbon Trust, IEA Wind). Search strings combined terms such as buoy wind measurement, floating LiDAR, motion compensation, attitude error, turbulence intensity, and offshore wind. The selected literature captures the technological evolution from early mechanical anemometry to modern remote-sensing instrumentation. Selection criteria prioritized studies that (1) addressed wind measurements collected from floating platforms, (2) provided explicit descriptions of motion-error mechanisms or compensation schemes, and (3) presented empirical validation or quantitative performance analysis. The review references 95 key contributions, including peer-reviewed articles and authoritative technical reports, to provide a representative overview of the state-of-the-art.

## 3. Buoy-Based Wind Measurement Systems and Their Motion Characteristics

### 3.1. Types and Measurement Principles of Buoy-Mounted Anemometers

Wind sensors deployed on marine buoys have changed considerably over the past seven decades [14]. The earliest instruments were purely mechanical; today, sonic anemometers and scanning LiDARs dominate new installations. Each technology rests on a different physical principle, and that principle largely determines how sensitive the sensor is to platform motion—and, consequently, how much effort must go into compensation [24]. The subsections below describe the three main categories in roughly the order they entered operational service.

#### 3.1.1. Cup and Propeller Anemometers

Cup and propeller anemometers are the workhorses of early marine meteorology [29,30]. Their operating principle is mechanical: wind spins a rotor—three or four hemispherical cups or a helical propeller—and the rotation rate is converted to wind speed through a calibration curve. Although these sensors are valued for their robustness and low cost, they exhibit significant limitations in dynamic environments. Their mechanical inertia introduces dynamic response lag, preventing accurate capture of high-frequency wind fluctuations, and they are prone to the well-documented “overspeeding” phenomenon in turbulent flows, which leads to systematic overestimation of mean wind speed [31,32].

Platform motion adds further complications. Early wind-tunnel experiments by Sanuki and Kimura under static inclination conditions demonstrated that cup anemometer readings are highly sensitive to tilt, with errors dependent on the tilt direction relative to the wind vector. Specifically, pitching was found to inflate readings by up to 20%, while rolling could suppress them by as much as 25% [33,34]. These experiments provide important baseline insight into directional sensitivity to inclination. However, pitch and roll are defined in the buoy’s body-fixed coordinate system, and in operational environments, the effective inclination relative to the wind varies continuously with the buoy’s instantaneous heading (yaw) and motion. As a result, the angle of attack experienced by the sensor becomes time-dependent—a feature that modern compensation methods address through continuous three-dimensional coordinate transformations (as discussed in Section 5). In addition, prolonged exposure to the marine environment results in bearing wear, corrosion, and salt crystallization, progressively degrading measurement accuracy and long-term reliability [29,35,36]. These limitations—intrinsic dynamic-response problems compounded by motion sensitivity—eventually motivated the development of non-mechanical alternatives for buoy-based wind measurement.

#### 3.1.2. Ultrasonic Anemometers

Ultrasonic anemometers were developed precisely to overcome the limitations of mechanical sensors and operate based on the acoustic time-of-flight (TOF) principle. Pairs of transducers face each other across a short open path; each transducer alternately transmits and receives ultrasonic pulses. Wind along the path speeds up sound traveling downwind and slows it traveling upwind. By measuring the transit-time difference, the instrument infers the wind component along that axis. With three such paths arranged in different orientations, a full three-dimensional wind vector can be reconstructed at sampling rates of tens of hertz [37,38,39].

As a result, ultrasonic anemometers respond almost instantaneously to velocity changes and require little maintenance [14,40,41]. These advantages have made them the default choice on modern research buoys. Even so, they are not immune to motion effects. Although ultrasonic anemometers avoid the inertia and overspeeding issues of mechanical sensors, they remain sensitive to platform motion. In addition to sensor-geometry-related flow distortion (shadowing) [42], the fundamental motion-induced error pathways—such as orientation-related projection errors and relative-wind contamination—are physically similar to those affecting mechanical anemometers. A key advantage of ultrasonic systems, however, is that they directly measure three orthogonal wind-velocity components at high temporal resolution. This three-dimensional information provides a stronger basis for coordinate transformation and kinematic correction, enabling more effective motion-compensation strategies than are typically possible with conventional mechanical sensors.

#### 3.1.3. Doppler Wind LiDAR

The arrival of Doppler LiDAR—particularly in the offshore wind-energy sector—marked a shift from point measurements to remote sensing of the wind field [26,43]. A LiDAR emits laser pulses (or, in continuous-wave designs, a focused beam) into the atmosphere. Aerosol particles carried by the wind scatter a small fraction of the light back toward the instrument. The Doppler shift in this backscattered light reveals the component of wind velocity along the beam direction, commonly called the line-of-sight or radial velocity. To reconstruct a horizontal wind vector or a vertical profile, the LiDAR scans its beam through multiple orientations—conical sweeps (e.g., Velocity–Azimuth Display (VAD) and Discrete Beam Positioning (DBS)) or other patterns—and combines the radial measurements geometrically [44,45,46,47]. This remote-sensing capability is a major advantage: the measurement volume can be placed tens or hundreds of meters away from the buoy, avoiding the flow disturbance that affects mast-mounted sensors [43].

However, this same remote-sensing principle makes LiDAR exceptionally sensitive to platform motion [48]. Even small buoy tilts, oscillations, or translations result in beam misalignment and geometric distortion of the measurement volume, leading to range-gate mixing and degraded wind-profile retrievals [5,17]. The associated error mechanisms are substantially more complex than those affecting point-based sensors and remain a major focus of ongoing research.

In addition to motion-related effects, LiDAR performance is inherently influenced by atmospheric scattering conditions. The availability and distribution of aerosol particles determine the strength and quality of the backscattered signal; consequently, low aerosol concentrations (e.g., in pristine open-ocean air) can reduce the carrier-to-noise ratio (CNR) and degrade data availability [26]. Furthermore, meteorological phenomena such as heavy precipitation or thick fog can attenuate the laser beam and introduce additional signal variability [49]. While distinct from kinematic motion errors, these atmospheric limitations are critical factors that influence the overall reliability of floating LiDAR systems in offshore environments.

Table 1 summarizes the fundamental measurement principles of commonly used buoy-mounted wind sensors and highlights their intrinsic sensitivity to platform motion, which provides context for the motion-induced error mechanisms discussed in Section 4.

### 3.2. Typical Types of Buoy Platforms

A buoy platform used to support anemometers is a complex marine engineering structure whose design geometry, dimensions, and mooring configuration collectively determine its dynamic response to ocean waves, and, consequently, the magnitude and characteristics of motion-induced measurement errors. Buoy platforms designed for wind-speed observations generally fall into three primary categories.

#### 3.2.1. Moored Meteorological Buoys

Moored meteorological buoys—such as the 3 m and 10 m disk-type buoys widely deployed by the U.S. National Data Buoy Center (NDBC) [18,50,51]—are the most common platforms used in marine observation networks. Fixed at designated locations using single-point or multi-point mooring systems, they are capable of long-term, stationary, and continuous measurements. Their motion characteristics are governed by the hydrodynamic behavior of the buoy and its coupled interaction with the mooring system [52]. The buoy’s size, geometry (e.g., disk, spherical, and cylindrical), and mass distribution determine its frequency-dependent Response Amplitude Operator (RAO) under waves of varying periods [53].

#### 3.2.2. Drifting Buoys

Drifting buoys are typically smaller, lighter, and more cost-effective than moored platforms. Deployed without a mooring system, they follow surface currents while transmitting meteorological and positional data via satellite systems [54]. The absence of mooring interactions allows GNSS-derived position and velocity measurements to directly capture bulk platform translation, which can be advantageous for correcting low-frequency drift motion. However, drifting buoys are continuously influenced by wave forcing, windage on the surface float, and current shear, resulting in irregular and non-stationary translational and rotational motions. Most drifters carry only compact meteorological sensors and positioning units (e.g., GNSS), without dedicated multi-axis inertial sensors or motion-reference systems, as documented in existing surface-drifter designs and air–sea interaction studies [55]. While GNSS data can assist in correcting large-scale translational velocity, the lack of continuous attitude and angular-rate measurements limits accurate six-degrees-of-freedom motion compensation.

As a result, wind measurements from drifting buoys are primarily suited for large-scale climatological analyses and air–sea interaction studies, rather than high-precision wind-resource assessment or turbulence characterization [56].

#### 3.2.3. Floating LiDAR Systems (FLS)

These specialized platforms are developed for one purpose: to carry a scanning LiDAR while keeping it as stable as possible [26,57]. Because LiDAR measurement quality degrades rapidly with tilt, FLS designers favor hull forms with inherently low angular response—deep-draft spars, tension-leg configurations, or multi-column semi-submersibles. Motion performance, particularly the transfer function from wave height to platform inclination, is a primary design metric and must be validated through wave-tank testing and open-ocean campaigns before commercial deployment [58].

**Figure 1:**
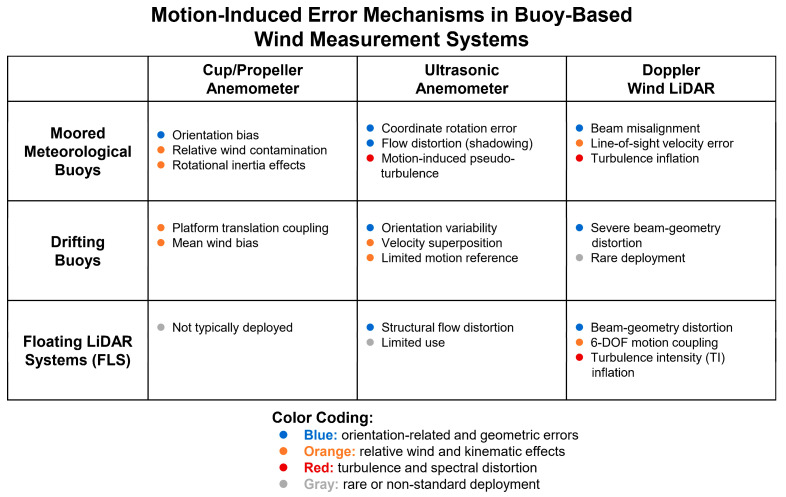
Matrix-based classification of motion-induced error mechanisms in buoy-based wind measurement systems. The figure maps dominant error sources across different platform types (moored meteorological buoys, drifting buoys, and floating LiDAR systems) and anemometer categories (cup/propeller anemometers, ultrasonic anemometers, and Doppler wind LiDAR). Error mechanisms are grouped into orientation-related and geometric errors, kinematic and relative-wind effects, turbulence-related spectral distortions, and rare or non-standard deployment scenarios, highlighting how platform motion characteristics and sensor principles jointly determine the dominant error pathways.

To provide an overview of how motion-induced error mechanisms depend jointly on platform type and sensor principle, Figure 1 presents a matrix-based classification of dominant error sources across buoy configurations and anemometer categories. Rather than depicting a process flow, this figure serves as a taxonomy that highlights the structural correspondence between platform motion characteristics, sensor response mechanisms, and the resulting wind-measurement errors, thereby guiding the detailed discussions in Section 4.

### 3.3. Buoy Motion Characteristics

**Figure 2:**
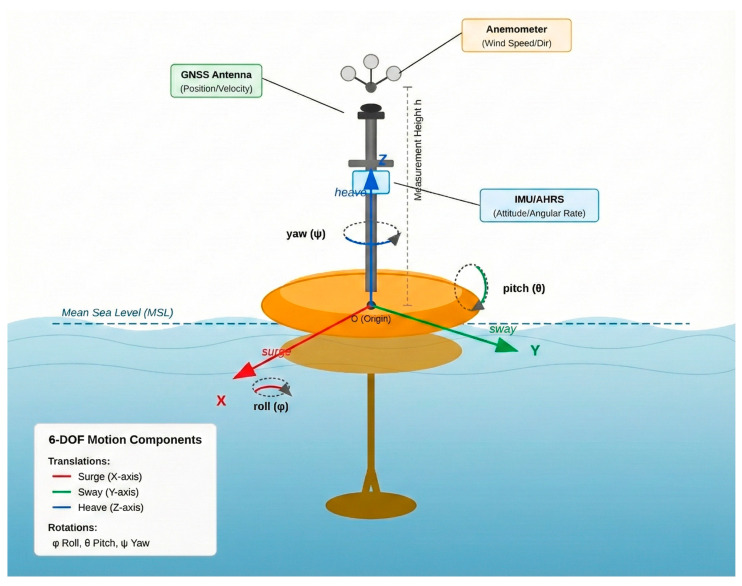
Schematic of a buoy-based wind measurement system illustrating the six-degree-of-freedom (6-DOF) motion components and sensor configuration. The coordinate system origin is defined at the buoy hull center, with translational motions (surge, sway, and heave) and rotational motions (roll φ, pitch θ, and yaw ψ) indicated.

In real ocean environments, buoy motion is driven by the combined and interacting forces of wind, waves, and currents, which produce a complex hydrodynamic response. This motion is conventionally decomposed into six degrees of freedom (6-DOF): three translational components—surge, sway, and heave along the x, y, and z axes—and three rotational components—roll, pitch, and yaw about their respective axes [12]. As illustrated in Figure 2, these six degrees of freedom collectively describe the full spectrum of buoy motions under combined wind–wave–current forcing.

Waves constitute the dominant source of buoy motion across a range of low-frequency bands relevant to ocean surface dynamics [59,60]. Due to the stochastic and irregular nature of ocean waves, buoy motion does not follow simple harmonic behavior but instead exhibits a broadband spectral response. In most sea states, wave energy is concentrated near the dominant wave frequency, typically within 0.05–0.5 Hz, which primarily induces roll, pitch, and heave motions with periods comparable to those of the incident waves [18,21]. In addition to this dominant band, other motion components coexist within the low-frequency regime, including relatively higher-frequency responses associated with short-period breaking waves and local structural interactions, as well as lower-frequency motions such as long-period swell and slow-drift responses induced by mooring dynamics. These components, although still well below typical sensor sampling limits, collectively produce multi-scale and non-stationary variations in buoy attitude [61,62].

Wind and surface currents primarily drive the buoy’s low-frequency motion and quasi-static offset [15]. Aerodynamic loading on the above-water structure, together with hydrodynamic forces acting on the submerged hull and mooring system, generates a persistent mean tilt and drift displacement [63,64,65]. Although these attitude variations evolve slowly in time, they represent a significant source of systematic wind-speed bias by inducing sustained sensor-orientation errors [15]. A comprehensive characterization of buoy motion, therefore, requires consideration of both wave-frequency–band dynamic responses and wind–current-driven quasi-static offsets.

Although moored buoys, drifting buoys, and floating LiDAR systems are all subject to six-degree-of-freedom motion, their dominant hydrodynamic characteristics differ substantially [66], leading to distinct error signatures and, consequently, different priorities in motion-compensation strategy design.

Moored meteorological buoys exhibit wave-dominated oscillatory motion coupled with mooring-induced low-frequency responses, resulting in persistent orientation bias and low-frequency velocity contamination [67]. Accordingly, compensation strategies for moored platforms prioritize accurate coordinate transformation and correction of quasi-static attitude offsets [68].

Drifting buoys, in contrast, experience unconstrained translational motion driven by surface currents and windage, with continuously varying headings [69]. Their error characteristics are dominated by relative-wind contamination and frame-velocity coupling, making kinematic correction and statistical filtering more relevant than full attitude stabilization [55].

Floating LiDAR systems represent a third regime, in which measurement accuracy is highly sensitive to even small residual platform motions because of the geometric amplification inherent in remote-sensing retrievals [26]. As a result, compensation strategies for FLS emphasize high-resolution attitude estimation, wave-response modeling, and motion–wind signal decoupling techniques.

These platform-dependent differences underscore that effective motion compensation must be tailored to the dominant motion modes and measurement principles of each platform type, rather than applied uniformly across systems.

## 4. Error Mechanisms in Wind Measurements Induced by Attitude Variations

As a floating observational platform situated on a continuously evolving sea surface, a buoy does not directly record the undisturbed atmospheric wind. Rather, the measured signal represents a superposition of the true atmospheric flow and the buoy’s six-degree-of-freedom (6-DOF) motion. Driven by the combined action of waves, wind, and currents, the buoy undergoes persistent changes in position and attitude. These variations introduce motion-related artifacts into wind-sensor outputs through multiple physical mechanisms, thereby producing observational errors. Importantly, these mechanisms often interact and occur simultaneously, forming the primary obstacle to obtaining accurate wind measurements from floating platforms.

**Figure 3:**
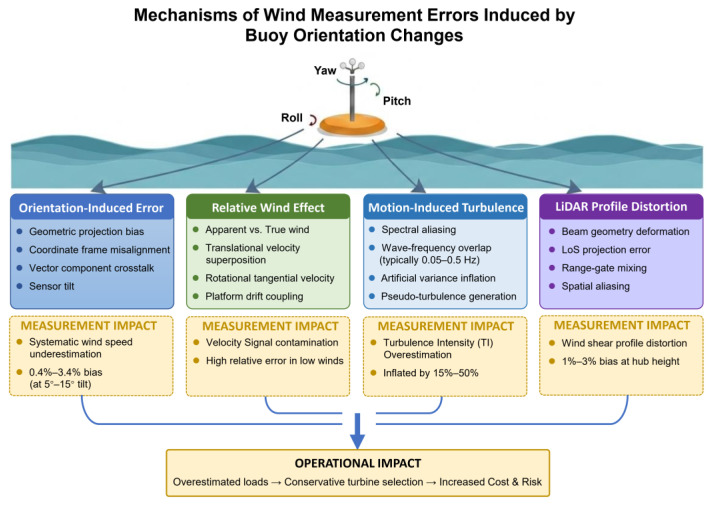
Overview of wind-measurement error mechanisms induced by buoy platform motion, including orientation-induced geometric bias, relative-wind contamination, motion-induced pseudo-turbulence, and LiDAR profile distortion. These effects can lead to systematic wind-speed bias (≈0.4–3.4%), turbulence-intensity inflation (typically 15–50%), and hub-height wind-profile deviations of about 1–3%.

As illustrated in Figure 3, external environmental forcing coupled with 6-DOF buoy motion establishes a clear causal chain linking platform dynamics to several categories of wind-measurement error. This section analyzes four dominant mechanisms, presented in order of increasing complexity:

(1) orientation-induced errors arising from platform tilt,
(2) relative-wind contamination introduced by translational and rotational motion,
(3) pseudo-turbulence driven by high-frequency oscillatory motion,
(4) LiDAR-specific profile distortion caused by beam misalignment and range-gate mixing.

The following subsections provide a mechanistic classification along with a detailed discussion of each attitude-induced error pathway.

### 4.1. Orientation-Induced Measurement Error

Orientation-induced errors arise from buoy tilt motions—primarily pitch and roll—that directly alter the spatial alignment of the wind speed sensor [42,70]. As the sensor coordinate system rotates along with the platform, a consistent mismatch arises between the measured wind vector and the true Earth-referenced wind vector.

#### 4.1.1. Coordinate Transformation Framework

Let the true wind-velocity vector in the Earth-fixed coordinate system (east–north–up, ENU) be denoted as

$$V_{true}^{E}=\left[\begin{array}{c} u\\ v\\ w \end{array}\right]$$

where u, v, and w represent the eastward, northward, and vertical wind components, respectively.

Due to platform rotation, the sensor measures wind in a body-fixed coordinate system. The transformation between these frames is governed by the direction cosine matrix (DCM), constructed from three sequential Euler angle rotations—yaw (ψ), pitch (θ), and roll (φ):

$$R=R_{z}(\psi )\cdot R_{y}(\theta )\cdot R_{x}(\varphi )$$

where the individual rotation matrices are

$$R_{z}(\psi )=\left[\begin{array}{ccc} \cos\psi & \sin\psi & 0\\ -\sin\psi & \cos\psi & 0\\ 0 & 0 & 1 \end{array}\right]$$

$$R_{y}(\theta )=\left[\begin{array}{ccc} \cos\theta & 0 & -\sin\theta \\ 0 & 1 & 0\\ \sin\theta & 0 & \cos\theta \end{array}\right]$$

$$R_{x}(\varphi )=\left[\begin{array}{ccc} 1 & 0 & 0\\ 0 & \cos\varphi & \sin\varphi \\ 0 & -\sin\varphi & \cos\varphi \end{array}\right]$$

The wind vector measured in the sensor’s body-fixed frame is related to the true wind by

$$V_{measured}^{B}=R\cdot V_{true}^{E}$$

Consequently, to recover the true wind from measured data, the inverse transformation must be applied:

$$V_{true}^{E}=R^{-1}\cdot V_{measured}^{B}=R^{T}\cdot V_{measured}^{B}$$

#### 4.1.2. Horizontal Wind Speed Underestimation

For the simplified case of pure pitch (θ) with no roll or yaw, the measured horizontal wind speed $V_{h,meas}$ relates to the true horizontal wind speed $V_{h,true}$ by

$$V_{h,meas}=V_{h,true}\cdot \cos\theta$$

Since cosθ≤1, this geometric projection produces a systematic low bias. For small tilt angles ($\theta <10^{{}^{\circ}}$), the relative error can be approximated as

$$\frac{∆V_{h}}{V_{h}}\approx 1-\cos\theta \approx \frac{\theta^{2}}{2}$$

For example, a 5° tilt introduces approximately 0.4% underestimation, while a 15° tilt (common under energetic sea states) produces approximately 3.4% underestimation [19,71].

#### 4.1.3. Vector Component Crosstalk

When the full rotation matrix is applied, cross-coupling occurs between wind-velocity components. Expanding the transformation for the case of combined pitch and roll—under the assumptions of small angles and negligible yaw—yields

$$u_{meas}\approx u-\omega \cdot \theta$$

$$v_{meas}\approx v+\omega \cdot \varphi$$

$$w_{meas}\approx w+u\cdot \theta -v\cdot \varphi$$

These expressions indicate that the true vertical wind component w leaks into the measured horizontal components $u_{meas}$ and $v_{meas}$, while horizontal wind components contaminate the measured vertical velocity $w_{meas}$. This vector-component crosstalk is particularly problematic for turbulence and flux calculations, where accurate estimation of the vertical wind component is essential [71,72].

#### 4.1.4. LiDAR Beam Geometry Distortion

For Doppler wind LiDARs employing conical scanning (e.g., VAD or DBS methods), platform tilt distorts the intended beam geometry. If the designed half-cone angle is α and the platform tilts by an angle θ, the actual elevation angles of individual beams deviate from their nominal value. For a four-beam DBS configuration, the effective elevation angles become

$$\alpha_{eff,i}=\alpha \pm \theta \cdot \cos\left(\varphi_{i}-\varphi_{tilt}\right)$$

where $\varphi_{i}$ is the azimuth of beam *i* and $\varphi_{tilt}$ is the direction of the platform. This asymmetry violates the geometric assumptions underlying standard wind-field inversion algorithms, introducing errors in both horizontal wind-speed retrievals and wind-direction estimates [5,22,48].

### 4.2. Relative Wind Effect Induced by Platform Motion

Beyond static tilt, the dynamic motion velocity of the buoy platform represents another major source of measurement error [17,72]. A wind-speed sensor measures airflow velocity relative to itself (the apparent wind), whereas the quantity of interest is the wind velocity relative to the Earth-fixed reference frame (the true wind). This fundamental difference implies that any translational or rotational motion of the platform generates a relative-wind component, thereby introducing bias and contaminating the measured signal.

#### 4.2.1. True Wind Recovery Equation

According to the principle of relative motion, the true wind velocity is obtained by subtracting the platform-motion velocity from the measured apparent wind:

$$V_{true}=V_{apparent}-V_{platform}$$

This vector subtraction must be performed in a common reference frame—typically the Earth-fixed ENU system. Therefore, the apparent wind measured in the buoy’s body frame must first be transformed using the rotation matrix **R** described in Section 4.1:

$$V_{true}^{E}=R^{T}\cdot V_{apparent}^{B}-V_{platform}^{E}$$

#### 4.2.2. Platform Motion Velocity Decomposition

The velocity of platform motion at the sensor position consists of two parts: the translational movement of the buoy as a whole and the additional tangential velocity generated by platform rotation. This relationship can be expressed as

$$V_{platform}=V_{trans}+\omega \times r$$

where

- $V_{trans}=\left[\begin{array}{c} u_{x}\\ u_{y}\\ u_{z} \end{array}\right]$ represents the translational velocity components of the buoy in a body-fixed Cartesian coordinate system, with $u_{x}$, $u_{y}$, and $u_{z}$ corresponding to surge (x-direction), sway (y-direction), and heave (z-direction) motions, respectively. These translational velocities are typically derived from GNSS-measured velocities expressed in an Earth-fixed reference frame and subsequently transformed into the buoy-fixed frame using the attitude information provided by the IMU.
- $\omega =\left[\begin{array}{c} p\\ q\\ r \end{array}\right]$ denotes the angular velocity vector comprising roll rate p, pitch rate q, and yaw rate r, measured by IMU gyroscopes.
- $r=\left[\begin{array}{c} x_{s}\\ y_{s}\\ z_{s} \end{array}\right]$ is the lever-arm vector from the buoy’s rotational center to the wind sensor.

The cross-product term explicitly accounts for the kinematic coupling between translational and rotational motions. Expanding this term yields

$$\omega \times r=\left[\begin{array}{c} q\cdot z_{s}-r\cdot y_{s}\\ r\cdot x_{s}-p\cdot z_{s}\\ p\cdot y_{s}-q\cdot x_{s} \end{array}\right]$$

The *z*-axis is defined as the local vertical normal to the mean sea surface, consistent with standard marine engineering practice. Here, the coordinate system is defined such that the *x*-axis corresponds to geographic north for moored buoys. For drifting platforms, the *x*-axis is instead aligned with the buoy’s body-fixed forward direction and is not necessarily coincident with the mean drift direction. This distinction ensures that the motion decomposition remains consistent across different deployment modes.

#### 4.2.3. Magnitude of Motion-Induced Contamination

The magnitude of motion-induced velocity contamination is governed by several factors, including sea-state energy, buoy geometry, and sensor installation configuration. Observations from moored buoy campaigns show that platform-induced velocities can represent a non-negligible portion of the true wind field under energetic conditions. For example, Pond (1968) reported motion velocities on the order of 0.5–1.0 m/s during moderate sea states—a level that can introduce substantial bias when ambient winds are weak (typically < 5 m/s) [21]. More recent assessments of floating LiDAR platforms have recorded heave motion exceeding 2 m/s, with horizontal surge and sway reaching 1–2 m/s during storm events where significant wave height exceeds 4 m [9,13].

The rotational component, represented by ω×r, is controlled jointly by the platform’s angular velocity and the lever-arm distance from the center of rotation to the sensor. For typical mounting heights of 3–5 m above the water surface, angular rates of 5–10°/s—which commonly occur under moderate wave forcing—can generate tangential velocities of approximately 0.3–0.9 m/s at the sensor position [19,73]. It should be emphasized that the relative significance of these motion-induced velocities depends strongly on the ambient wind conditions and prevailing sea-state regimes. While high wave heights are often associated with strong winds in sea-wind-dominated environments, scenarios characterized by energetic swell and comparatively weak local winds can occur, particularly in tropical and subtropical regions or during swell-dominated conditions. In such cases, motion-induced velocities may constitute a substantial fraction (20–50%) of the measured wind signal, leading to pronounced relative errors despite moderate absolute motion amplitudes [20,74]. This context dependence highlights the importance of interpreting motion-contamination magnitude in terms of relative error, rather than absolute velocity alone.

#### 4.2.4. Heave-Induced Profile Mixing

For wind-profile observations, vertical heave motion causes the sensor to move through multiple atmospheric layers during each averaging interval. Assuming the wind field follows a power-law shear profile

$$U(z)=U_{ref}{\left(\frac{z}{Z_{ref}}\right)}^{\alpha }$$

where the shear exponent α lies in the range of 0.10–0.14 over ocean surfaces [43], a buoy experiencing heave with amplitude $A_{h}$ will sample an oscillating measurement height given by

$$z(t)=z_{0}+A_{h}\sin\left(\omega_{h}t\right)$$

with $z_{0}$ denoting the nominal sensor height. As a result, the recorded wind speed corresponds to a time-averaged value over the vertical span $z_{0}-A_{h}$ to $z_{0}+A_{h}$, rather than the intended fixed elevation. This vertical sampling smearing introduces bias into both the estimated shear exponent and the extrapolated hub-height wind speed. 

Vertical extrapolation of wind speed is commonly performed using either the logarithmic wind profile or empirical power-law formulations. Under near-neutral atmospheric stability, the logarithmic profile derived from Monin–Obukhov similarity theory (MOST) remains widely applicable within the atmospheric surface layer and is frequently used for wind resource assessment at turbine-relevant heights [8]. The power-law profile provides a convenient empirical alternative and is often adopted in engineering practice when detailed stability or roughness information is unavailable. In offshore environments, where surface roughness is low and stability conditions can vary, both approaches represent approximations, and their accuracy depends on atmospheric stratification and measurement context rather than on a strict height threshold.

Kelberlau and Mann (2022) reported that heave amplitudes of 1–2 m can produce wind-speed deviations of approximately 1–3% in hub-height wind-speed estimates at a typical turbine hub height of 100 m [17].

### 4.3. Motion-Induced Turbulence

Beyond its impact on mean wind-speed estimation, buoy motion can substantially distort the fluctuating component of the wind signal, thereby contaminating turbulence measurements [48,75]. This contamination arises because platform motion introduces artificial velocity fluctuations that overlap with the frequency band used for atmospheric turbulence analysis and, in many cases, obscure the true atmospheric turbulence spectrum.

#### 4.3.1. Spectral Overlap and Aliasing

The power spectral density of wind-speed fluctuations can be decomposed into atmospheric-turbulence and motion-induced components:

$$S_{vv}(f)=S_{atm}\left(f\right)+S_{motion}(f)+2\cdot C_{atm\text{-}motion}(f)$$

where $S_{atm}\left(f\right)$ is the true atmospheric-turbulence spectrum, $S_{motion}(f)$ is the platform-motion-induced velocity spectrum, and $C_{atm\text{-}motion}(f)$ represents the cross-spectral density. The cross-term is often assumed to be small as a first-order analytical approximation; however, in reality, atmospheric turbulence can contribute to platform motion (via aerodynamic forcing) and introduce some degree of coupling.

Ocean-wave energy is concentrated in the frequency band 0.05–0.3 Hz (corresponding to wave periods of 3–20 s), which lies well below typical sensor sampling rates but overlaps significantly with the energy-containing range of atmospheric boundary-layer turbulence [9,17,75]. This spectral overlap makes it statistically difficult to distinguish true turbulent fluctuations from motion-induced artifacts in the measured signal.

#### 4.3.2. Turbulence Intensity Overestimation

Turbulence intensity (TI) is defined as

$$TI=\frac{\sigma_{u}}{\bar{U}}$$

where $\sigma_{u}$ is the standard deviation of the along-wind velocity component and $\bar{U}$ is the mean wind speed over the averaging period (typically 10 min). 

When motion-induced fluctuations are present, the measured variance becomes

$$\sigma_{u,meas}^{2}=\sigma_{u,atm}^{2}+\sigma_{u,motion}^{2}$$

Consequently, the measured TI is biased high:

$${TI}_{meas}=\frac{\sqrt{\sigma_{u,atm}^{2}+\sigma_{u,motion}^{2}}}{\bar{U}}>{TI}_{true}$$

The relative overestimation can be expressed as

$$\frac{{TI}_{meas}-{TI}_{true}}{{TI}_{true}}=\sqrt{1+(\frac{\sigma_{u,motion}}{\sigma_{u,atm}}{)}^{2}}-1$$

Field studies have reported TI overestimations ranging from 15% under moderate sea states to more than 50% under energetic conditions [9,22,35,75]. Thiébaut et al. (2024) experimentally demonstrated that uncorrected floating LiDAR measurements exhibited TI values approximately 1.3–1.5 times higher than fixed reference measurements [9].

#### 4.3.3. Implications for Offshore Wind Applications

TI overestimation has significant engineering consequences:

1. Fatigue load assessment

Turbine design standards (e.g., IEC 61400-1) use TI to determine fatigue damage-equivalent loads. Overestimated TI leads to conservative load predictions and may result in unnecessarily robust—and costly—turbine components.

2. Turbine selection

Site-specific TI is used to classify wind turbine classes (A, B, or C) according to environmental conditions. A biased TI estimate may trigger the selection of a higher-class, more expensive turbine than required.

3. Wake modeling and farm layout optimization

TI is a key input to engineering wake models. Errors in turbulence intensity (TI) estimation can propagate into wake-recovery modeling and degrade the accuracy of wind-farm array-efficiency evaluations.

4. Gust and extreme-event misclassification

Motion-induced velocity fluctuations may be falsely identified as extreme gusts, leading to biased extreme-load calculations and unnecessarily conservative structural design requirements [22,35].

### 4.4. LiDAR-Induced Profile Distortion

For floating LiDAR systems (FLS), the remote-sensing measurement architecture inherently increases sensitivity to platform motion, giving rise to a distinct class of wind-profile distortion errors [17]. LiDAR systems infer wind profiles through volumetric scanning and geometric reconstruction. Measurement accuracy, therefore, depends critically on the stability of the scanning geometry and the consistency of the optical line-of-sight (LoS) configuration [47]. Six-degree-of-freedom (6-DOF) platform motion perturbs this geometry through multiple coupled mechanisms.

(i) Heave-induced sampling-height modulation

Vertical heave motion introduces continuous variation in the instantaneous sampling height of the LiDAR probe volume. It should be emphasized that this effect does not imply a physical mast of comparable height; rather, the LiDAR instrument—typically mounted only a few meters above the sea surface—remotely interrogates wind velocities at nominal heights (e.g., 100 m) through beam propagation. Platform heave therefore causes the effective probing height to oscillate around the target level (e.g., ≈98–102 m), resulting in vertical profile mixing.

This height modulation leads to a time-weighted averaging of wind velocities over a finite vertical interval, rather than sampling at a single fixed elevation. In addition, range-dependent variations in signal-to-noise ratio modify the weighting of the returned Doppler signal, increasing retrieval variance and degrading the resolution of shear and turbulence statistics [5,9,57].

(ii) Pitch- and roll-induced scan-geometry distortion

Angular motions of the platform tilt and deform the scanning cone, causing rotation of LoS vectors and geometric distortion of VAD sampling surfaces [22,24]. Instead of interrogating a constant-elevation annulus, the LiDAR samples a time-varying three-dimensional manifold. This geometric deformation produces spatial aliasing, whereby the reconstructed Doppler field represents a weighted combination of wind velocities from multiple heights and lateral offsets, reducing sensitivity to fine-scale turbulence and vertical shear gradients [17].

These distortion effects are particularly pronounced under spatially heterogeneous flow conditions—such as coastal boundary layers or nearshore environments—where vertical and horizontal wind gradients are strong. In such cases, the superposition of multi-level velocity contributions can lead to systematic bias in reconstructed wind profiles, even when mean platform motions are moderate [76].

## 5. Attitude Error Compensation Techniques and Their Development

Building on the mechanistic framework outlined in Section 3, motion-error mitigation requires compensation strategies that directly target the individual physical pathways through which buoy dynamics perturb wind measurements. Since orientation misalignment, relative-wind contamination, motion-induced pseudo-turbulence, and LiDAR profile distortion arise from distinct yet coupled dynamical processes, a single universal correction method is insufficient to suppress all error modes across operational conditions.

Accordingly, a broad family of compensation approaches has evolved, encompassing geometric attitude corrections, rigid-body motion subtraction, physics-based kinematic models, multi-sensor fusion architectures, hydrodynamic RAO-driven response estimation, and emerging data-driven inference algorithms. Although heterogeneous in formulation, these methods converge on the same objective: explicit decoupling of the atmospheric wind vector from motion-induced velocity components embedded within raw measurements.

The trajectory of methodological development reflects increasing sophistication in both physical understanding and computational capability. Progress in IMU/GNSS sensor accuracy, signal-processing bandwidth, numerical hydrodynamics, and deep-learning frameworks has enabled progressively improved separation of true wind signals from platform-motion artifacts. Together, these advancements establish the foundation for high-fidelity, resilient, and operationally scalable wind-measurement systems on floating platforms.

### 5.1. Physical-Model-Based Attitude Correction and Motion Compensation

As the most fundamental and physically interpretable class of compensation strategies, physical-model-based methods conceptualize buoy motion as a measurable rigid-body process and eliminate platform-induced velocity components from the “contaminated” wind record through explicit vector operations [68,72]. Serving as the conceptual foundation for most modern correction frameworks, these approaches are implemented through two core procedures: (i) coordinate-system transformation and (ii) platform-velocity subtraction [17].

Each component directly addresses a dominant error pathway identified in Section 4. Coordinate-frame transformation corrects orientation-induced bias associated with pitch/roll misalignment (Section 4.1), whereas velocity-vector subtraction suppresses relative-wind contamination generated by translational and rotational motion (Section 4.2). In combination, these operations provide a physically transparent, mathematically tractable, and computationally efficient basis upon which more advanced hybrid or data-driven compensation schemes are constructed.

(i) Coordinate-System Transformation

Coordinate-system transformation addresses geometric orientation errors caused by buoy pitch, roll, and yaw [72]. Using real-time attitude angles obtained from sensors such as an Inertial Measurement Unit (IMU), a rotation matrix is constructed to map the sensor’s body-fixed coordinate frame to an Earth-fixed frame (e.g., east–north–up) [19]. By left-multiplying the measured three-dimensional wind-velocity vector by the inverse of this matrix, the measurement is transformed into the Earth frame, yielding wind-velocity components that are independent of platform tilt [17]. Studies by Li et al. (2020) and Zhao et al. (2024) applied similar transformations and confirmed their effectiveness in correcting wind direction and horizontal wind-speed components [44,77].

(ii) Velocity-Vector Subtraction

Velocity-vector subtraction is then used to remove the relative-wind effect introduced by platform motion [68]. The relative wind speed measured by the anemometer, ${\vec{V}}_{measured}$, is the vector sum of the true wind velocity, ${\vec{V}}_{true}$, and the platform-motion velocity, ${\vec{V}}_{buoy}$. Thus, the true wind velocity is obtained through

$${\vec{V}}_{true}={\vec{V}}_{measured}-{\vec{V}}_{buoy}$$

The platform-motion velocity ${\vec{V}}_{buoy}$ comprises translational velocities (surge, sway, and heave) measured by GNSS, as well as tangential velocities computed from the cross-product of IMU-measured angular rates and the lever-arm vector representing the sensor’s installation location. In seminal work on air–sea flux measurements from moving platforms, Edson et al. (1998) emphasized the critical importance of such velocity compensation and established a framework that has since been widely adopted [68].

(iii) Limitations of Physical-Model-Based Methods

Despite their clear physical interpretability, straightforward implementation, and low computational cost—which collectively enable efficient real-time deployment on embedded systems—physical-model-based approaches exhibit several inherent limitations [17]:

- High dependence on attitude-sensor accuracy and synchronization.

Compensation performance relies strongly on the precision, sampling rate, and timing alignment of GNSS/IMU measurements. Under high-frequency wave forcing, sensor noise, communication latency, and inter-instrument desynchronization can propagate into the correction process, significantly reducing compensation effectiveness [17,24,72].

- Limited capability for nonlinear or coupled-error mitigation.

Rigid-body subtraction implicitly assumes linear motion behavior and therefore cannot adequately resolve motion-induced pseudo-turbulence, where frequency-domain interference between platform motion and atmospheric turbulence produces complex, nonlinear contamination patterns [9,17].

Thus, although physical approaches form the baseline layer of buoy motion compensation, achieving high-fidelity wind-field reconstruction in energetic sea states necessitates more advanced methodologies—such as those introduced in Section 5.2, Section 5.3, Section 5.4 and Section 5.5.

### 5.2. Multisensor Data Fusion and Attitude Estimation Techniques

Addressing the sensor-accuracy limitations identified in Section 5.1, the performance bottleneck of physical-model-based compensation algorithms largely stems from the accuracy and continuity of attitude information acquisition [11,62]. In the turbulent marine environment, a single attitude sensor—particularly low-cost Micro-Electro-Mechanical Systems (MEMS) IMUs—often fails to provide stable, drift-free, and high-precision six-degree-of-freedom motion measurements. These sensors are highly susceptible to noise, bias drift, and signal dropouts under energetic sea states [78]. To overcome these limitations, researchers have increasingly adopted multisensor data-fusion techniques that integrate complementary information sources to obtain more robust and accurate attitude estimates.

Among fusion strategies, the integration of IMU and GNSS remains the most classical and widely used approach [62]. IMU gyroscopes provide high-frequency (hundreds of hertz) angular-rate measurements capable of capturing the rapid platform dynamics, although their integrated outputs accumulate drift over time [79]. In contrast, GNSS—especially dual-antenna systems employing Real-Time Kinematic (RTK) or Precise Point Positioning (PPP) techniques—offers drift-free absolute position, velocity, and heading measurements, albeit at lower sampling rates (1–10 Hz) [62,80]. These two sensors therefore exhibit strong complementarities across both temporal and spectral domains [11], making their fusion particularly effective for representing the dual-scale buoy motion characteristics described in Section 3.3.

To exploit this complementarity, a variety of filtering algorithms have been developed. The most widely applied are the Kalman filter and its nonlinear extensions—the extended Kalman filter (EKF) and the unscented Kalman filter (UKF) [79]. These algorithms construct system-state and observation models and recursively estimate platform attitude and velocity through a prediction–correction cycle, using high-frequency IMU data for dynamic prediction and low-frequency but high-accuracy GNSS data for correction [81]. For example, Salcedo-Bosch et al. (2021) applied an adaptive unscented Kalman filter (RAUKF) to floating LiDAR measurements and achieved substantial improvement in turbulence-intensity accuracy [82].

Beyond Kalman-based approaches, complementary filtering has gained popularity due to its low computational cost and simplicity of implementation [83]. By combining high-pass and low-pass filtering to extract high-frequency dynamic information from gyroscopes and low-frequency attitude information from accelerometers, magnetometers, or GNSS, complementary filters produce full-bandwidth attitude estimates through weighted fusion [70].

Multisensor data fusion significantly enhances the accuracy, continuity, and robustness of buoy attitude estimation, thereby providing more reliable inputs for physical-model-based wind-speed compensation algorithms [62]. However, challenges remain. Parameter tuning—for example, specifying noise covariance matrices—often depends on empirical adjustment, and fixed-parameter filters may fail to maintain optimal performance under rapidly changing sea states [11,84]. These limitations indicate the requirement for state-dependent, adaptively tuned filtering schemes capable of responding to nonstationary marine conditions [82].

### 5.3. RAO-Based Wave-Response Modeling and Motion Correction for Buoy Platforms

Complementary to the multi-sensor fusion frameworks in Section 5.2, a key compensation methodology employs the platform’s hydrodynamic response behavior by utilizing RAO formulations for forward motion prediction and subsequent correction [80]. RAO-based modeling describes the frequency-dependent motion response of the buoy platform itself and is therefore applicable to buoy-based wind measurement systems in general, independent of the specific wind-sensing technology employed. In practice, this approach has become a core component of several international commercial certification frameworks, particularly in the context of floating LiDAR systems (FLS), where platform-motion characterization is critical [26,85].

The underlying principle is that, for a buoy of a given design, the amplitude of its motion response in each degree of freedom under unit-amplitude regular-wave excitation is a function of wave frequency. This frequency-dependent relationship, known as the Response Amplitude Operator (RAO), describes the linear hydrodynamic transfer characteristics that map incident wave energy to platform motion [12,60,86]. Directly linking the dual-scale wave forcing described in Section 3.3 to observable platform dynamics. RAO models are typically derived from high-fidelity scaled wave-basin experiments or by using advanced computational fluid dynamics (CFD) simulations [86,87].

Once established, the operational workflow of RAO-based compensation is conceptually straightforward. During field deployment, the in situ wave spectrum is measured in real time using onboard wave sensors such as accelerometers or wave radars. By inputting the measured wave spectrum into the RAO model, the six-degree-of-freedom motion-response spectrum can be predicted, and the corresponding attitude time series reconstructed [12,60,85]. These motion estimates can then be used as surrogate attitude inputs for the physical-model-based compensation techniques described in Section 4.1 to correct the observed wind speed.

The RAO-based strategy offers several important advantages, particularly from a system-level robustness perspective rather than absolute accuracy, and it is typically used in conjunction with, rather than as a replacement for, direct attitude measurements. Most notably, it provides a physics-based motion estimate that is independent of direct attitude-measurement sensors such as IMUs or GNSS receivers. In scenarios where IMU signals deteriorate, saturate, or temporarily fail under energetic wave forcing, RAO-based motion reconstruction can serve as a complementary or fallback information channel, thereby improving overall system resilience and fault tolerance.

It should be noted, however, that RAO-based approaches are not expected to outperform direct inertial measurements under strongly nonlinear or breaking-wave conditions [66,88]. Instead, their primary value lies in providing a consistent, physically constrained estimate of platform motion when direct measurements are degraded, incomplete, or unavailable.

Despite these benefits, RAO-based approaches also exhibit intrinsic limitations. Their accuracy depends strongly on precise knowledge of buoy structural parameters and the fidelity of the hydrodynamic model; manufacturing tolerances and long-term structural changes—including marine biofouling—can introduce model drift and degrade performance [89,90]. Moreover, RAO models are fundamentally rooted in linear wave theory and therefore become less accurate under strongly nonlinear or breaking-wave conditions. Finally, RAO models are often developed for specific sea states or representative spectra, limiting their adaptability when real-world conditions deviate significantly. These challenges have motivated recent developments in online estimation and adaptive RAO modeling techniques.

### 5.4. Cost–Benefit Feasibility and Operational Readiness of Motion-Compensation Methods

Motion compensation is essential for ensuring the accuracy and reliability of buoy-based wind measurements, where wave-induced platform motion can significantly contaminate observed signals. From an engineering perspective, the practical adoption of compensation methods depends not only on achievable accuracy but also on implementation cost, computational demand, interpretability, and certification readiness. Here, four major categories of motion-compensation strategies are compared in terms of their cost–benefit feasibility and operational maturity. 

Physical-model-based correction methods rely on rigid-body kinematics and classical mechanics to correct orientation-induced geometric errors and relative-wind contamination. These approaches generally involve low to moderate implementation costs and impose minimal computational burden, making them well-suited for real-time onboard processing. Their transparent physical basis facilitates validation and certification, which explains their widespread adoption as the foundational layer in operational buoy systems [68,72]. However, their accuracy degrades under strongly nonlinear or highly energetic sea states, where simplified model assumptions may no longer hold.

Multi-sensor fusion techniques, typically integrating IMU and GNSS measurements through Kalman filtering, are best viewed as an extension of physical-model-based correction rather than a standalone alternative. Their primary objective is to improve the accuracy and long-term stability of motion estimates by mitigating sensor drift and noise. While sensor integration and filter tuning increase system complexity and cost, these methods remain compatible with real-time operation and are already well established in offshore engineering practice [91]. Consequently, multi-sensor fusion plays a critical enabling role by enhancing the reliability of motion inputs used in physical correction schemes.

RAO-based hydrodynamic modeling exploits the platform’s frequency-dependent wave-response characteristics to predict motion from measured wave spectra. Once developed, RAO models impose modest computational requirements and can operate in real time, providing a physics-based source of motion information independent of direct attitude sensors. This feature makes RAO-based approaches particularly valuable for floating LiDAR systems as a supplementary or fallback correction pathway. Nevertheless, their reliance on linear wave theory and platform-specific calibration limits accuracy under strongly nonlinear or rapidly evolving sea states [66].

AI and machine-learning-based compensation methods represent the most promising direction for addressing nonlinear and coupled error mechanisms, particularly motion-induced turbulence contamination. These methods can achieve superior correction performance by learning complex relationships between platform motion and measured wind signals. However, this accuracy potential comes at the cost of substantial data requirements, increased computational demand, and limited interpretability [92]. At present, these factors constrain real-time deployment on power-limited buoy platforms and pose challenges for certification, placing AI-based approaches closer to research and pre-operational demonstration than routine operational use.

In summary, physical-model-based correction combined with multi-sensor fusion defines the current operational baseline for buoy motion compensation, offering a robust balance between cost, reliability, and regulatory acceptance. RAO-based methods enhance system resilience in wave-dominated environments, while AI-driven techniques hold long-term potential for improved accuracy but remain limited by practical deployment and certification constraints.

### 5.5. Evolution of Attitude Compensation Techniques

**Figure 4:**
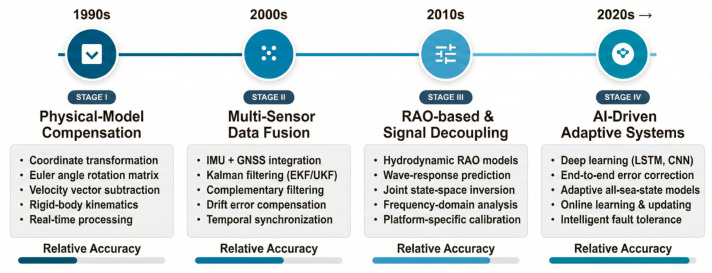
Schematic overview of the evolution of buoy attitude compensation techniques. The four stages represent the progression from fundamental physical-model-based corrections (1990s), through multi-sensor data fusion (2000s) and RAO-based hydrodynamic modeling (2010s), to emerging AI-driven adaptive compensation systems (2020s and beyond).

The development of buoy attitude-error mitigation techniques has evolved through several key stages, as illustrated in Figure 4.

Stage I: Fundamental Physical Corrections

Early methods, based on Newtonian mechanics, focused on platform motion correction through coordinate-frame rotation and platform velocity-vector subtraction. These basic techniques formed the foundation for modern compensation methods. 

Stage II: Sensor Fusion and Model-Assisted Approaches

The integration of multi-sensor fusion strategies, combining IMUs and GNSS data with Kalman filtering, significantly improved the stability and reliability of attitude estimates. RAO-based hydrodynamic-response modeling also played a key role in enhancing system robustness under varying sea states.

Stage III: Signal Decoupling and AI-Driven Methods

To address high-frequency spectral aliasing, signal-decoupling frameworks were introduced, and AI/machine-learning models emerged to provide nonlinear compensation by learning from motion-contaminated inputs to corrected outputs.

Stage IV: Adaptive All-Sea-State Compensation Systems (Future Outlook)

Research is moving toward adaptive systems capable of operating in all sea states, including extreme conditions. Future systems will integrate real-time RAO recalibration, adaptive filtering, and reinforcement learning to optimize compensation performance.

### 5.6. Comparative Performance of Attitude Compensation Methods

To provide a systematic assessment of the compensation techniques described above, this section evaluates their characteristics across several criteria, as shown in Table 2. It should be emphasized that these approaches are not designed to function independently. In real applications, modern systems often combine more than one method. For example, a practical configuration might use multi-sensor fusion to obtain reliable attitude information, apply physical-model compensation to remove basic motion effects, and then introduce AI-based techniques to handle remaining complex or nonlinear errors.

## 6. Major Challenges

The compensation framework described in Section 5 has advanced considerably over three decades, yet translating laboratory demonstrations and fair-weather validations into systems that perform reliably across all sea states remains an open problem. The obstacles are not purely technical; they also involve gaps in fundamental understanding and the absence of community-wide standards. Below, we discuss the principal challenges that currently limit operational deployment of buoy-based wind-measurement systems.

The foremost challenge concerns observational survivability and data reliability under extreme marine conditions. One of the most valuable use cases for buoy-based wind measurements is the acquisition of critical data during high-impact weather events such as typhoons and severe storms. Yet, it is precisely under these extreme sea states that current systems exhibit their greatest vulnerabilities. Violent wave impacts and intense six-degree-of-freedom motion can easily drive attitude sensors beyond their operational limits—for example, IMUs may saturate under excessive accelerations, and GNSS receivers may lose lock when antennas are intermittently submerged or experience large antenna-motion excursions [13,18]. For remote-sensing systems such as floating LiDAR, breaking-wave fronts, dense marine spray, and heavy precipitation can severely degrade the atmospheric optical path, causing attenuation or complete extinction of the laser backscatter signal [26,49]. These conditions result in intermittent or total loss of both attitude and wind-speed measurements—depriving motion-compensation algorithms of the continuous inputs they fundamentally require. This creates a critical paradox: when accurate wind-field observations are needed most, the capability to acquire them is at its weakest. Such critical data gaps represent one of the most serious operational challenges currently confronting buoy-based wind-observation systems.

Secondly, the physical mechanisms through which high-frequency and small-amplitude buoy motions contaminate turbulence measurements remain incompletely understood. Although numerous studies have demonstrated that platform motion leads to systematic overestimation of turbulence intensity (TI), the underlying processes are considerably more complex than simple linear superposition. In the higher-frequency range (>0.5 Hz), minute buoy vibrations, structural resonances, and nonlinear rocking motions may introduce additional complexity into turbulence measurements. However, these motions typically contain less energy than dominant wave-induced responses, and their influence becomes significant mainly when vibration frequencies overlap with sensor response bandwidths or when resonance effects are excited. The detailed mechanisms governing such high-frequency motion–turbulence interactions are not yet captured by any unified or broadly validated theoretical framework [17,22]. Existing compensation techniques, whether based on physical-model velocity subtraction or linear RAO-based response prediction, struggle to isolate true turbulent fluctuations from mixed motion–turbulence signals. This incomplete mechanistic understanding constitutes a fundamental theoretical limitation on improving higher-order turbulence statistics and directly constrains the utility of buoy-based observations for applications such as turbine fatigue-load assessment and air–sea flux estimation.

A further obstacle lies in the lack of standardization and engineering maturity across the current technical ecosystem. Compensation techniques have proliferated, but the community still lacks a universally accepted basis for comparing them. Although organizations such as the Carbon Trust have published recommended practice guidelines for FLS verification [26], these documents focus on mean wind speed and do not yet extend to turbulence metrics or to the broader universe of moored and drifting buoys. Individual studies adopt different reference systems—met masts of varying heights, fixed LiDARs, other buoys, and numerical reanalyses—and report performance using inconsistent metrics, making cross-study comparison difficult [83]. 

Furthermore, long-term sensor reliability must be balanced against cost, power consumption, and system complexity. High-grade IMUs and GNSS receivers impose significant cost and power burdens on buoys operating on batteries or limited solar energy [93]. Exposure to salt spray, high humidity, and biofouling degrades sensor performance over time; recalibrating or replacing instruments at sea is expensive and often impractical [89,94]. Achieving a practical balance among accuracy, reliability, and long-term operational stability within constrained cost and energy budgets remains a central engineering challenge in the design of buoy-based wind-measurement systems.

Taken together, these challenges define the current frontier. Progress will require coordinated advances across sensor hardware, platform design, algorithm development, and community standardization. The following section outlines research directions that, in our view, offer the greatest potential for closing these gaps.

## 7. Future Research Recommendations

Given the persistent influence of buoy attitude variability on wind-speed accuracy and the growing demand for high-fidelity wind-field measurements in deep-ocean observation and offshore wind-energy assessment, future research should converge toward developing an intelligent, robust, and standardized compensation architecture. Realizing this objective necessitates methodological innovation, coordinated advances in sensing hardware and platform engineering, and the establishment of unified performance-evaluation and certification systems. Four technical trajectories likely to drive future progress are summarized below.

(1) AI-Enabled Intelligent Sensing and Adaptive Compensation

A primary frontier is the deployment of AI-assisted inference models capable of learning complex motion–wind coupling dynamics. Conventional model-based methods remain limited when addressing strong nonlinearity and nonstationary sea states, whereas deep neural architectures offer high-dimensional representation capacity. Future development should emphasize end-to-end correction pipelines [95] that directly transform multi-sensor raw streams into bias-corrected wind-speed estimates. Heterogeneous feature fusion—including 6-DOF motion vectors, high-rate anemometer signals, wave spectral parameters, and image-based environmental cues—should be integrated via hybrid DL architectures (CNN–LSTM, GRU-attentive structures, or Transformer-based temporal encoders). Such models can learn implicit physical relationships without explicit turbulence-motion decoupling.

A promising subdirection involves data-driven mitigation of motion-induced TI inflation. AI models trained on combined atmospheric and motion labels can classify pseudo-turbulence components and reconstruct the true turbulent field [19,85]. Because annotated offshore datasets remain scarce, simulation-augmented training pipelines—involving high-fidelity CFD/LES turbulence synthesis, physics-informed neural networks (PINNs), and cross-domain transfer learning—will be essential for improving generalization across platforms and sea states.

(2) Adaptive and Robust Compensation Across All Sea States

A second research vector concerns adaptive compensation algorithms with sea-state-invariant robustness. Most existing RAO-based and fusion-based frameworks employ static parameters, constraining reliability in steep or multi-modal sea conditions. Future approaches should incorporate online RAO identification and real-time hydrodynamic model updating using concurrent wave-state estimation [87]. For state-space estimators, adaptive Kalman filtering and covariance tuning should be developed to auto-regulate process/measurement noise and maintain observability under variable platform motion or partial sensor degradation [82].

Operational resilience will further depend on redundant sensing and autonomous fault-tolerant logic. Systems should support seamless failover to backup inertial or predictive modes during GNSS outages, IMU drift events, or sensor fouling. Short-horizon motion prediction using autoregressive or neural forecasters may ensure continuity of wind reconstruction under intermittent data loss.

(3) Standardized Evaluation Frameworks and Benchmark Datasets

A third priority is the formulation of standardized validation frameworks and publicly accessible benchmark datasets. The rapid proliferation of compensation methods necessitates reference protocols comparable across platforms, sea states, and sensor configurations. Drawing on initiatives such as the Carbon Trust OWA roadmap, a unified certification standard for buoy-based and FLS wind measurement should specify as follows:

- test configurations and environmental regimes,
- reference instrumentation and cross-validation protocols,
- data-screening rules and reproducible workflows,
- multidimensional performance indicators (mean wind speed, TI, shear, spectral fidelity, and hub-height bias).

Parallel efforts should develop and release high-resolution open datasets containing synchronized wind, 6-DOF motion, and directional wave spectra. An integrated buoy–met mast–satellite–numerical model validation network will enable multi-source cross-verification and support the development of transferable, platform-agnostic compensation algorithms.

(4) Advanced Sensing Technologies and Intelligent Platform Design

A fourth technical path involves next-generation sensing hardware and motion-optimized platform engineering. Hardware improvements form the foundational layer, enabling high-quality correction. Priorities include high-stability IMUs with low bias drift, multi-band RTK-GNSS for centimeter-scale motion tracking, and corrosion-resistant sensors capable of long-duration deployment in harsh marine environments. On the platform side, active attitude-stabilization mechanisms—such as RAO-tunable hull geometries, dynamic ballast actuation, and fin-based control surfaces—offer engineered pathways to suppress pitch/roll excursions at the source.

In the long term, tight integration of algorithmic intelligence and cyber-physical design may lead to a software-defined wind-measurement architecture, wherein raw sensing, motion compensation, and quality control operate as a real-time autonomous loop. Such systems could deliver step-change improvements in accuracy, resilience, and autonomy for open-ocean wind-field observation.

## 8. Conclusions

Buoy-based platforms have become indispensable for observing sea-surface winds, yet the very mobility that makes them attractive also introduces persistent measurement errors. Through this review, we have sought to clarify how platform motion degrades wind observations and what remedies are currently available.

Our analysis leads to several findings. First, motion-induced errors are not monolithic; they arise from at least four distinct physical pathways—tilt-induced coordinate rotation, platform-velocity contamination, pseudo-turbulence from high-frequency oscillations, and LiDAR-specific geometric distortions—each requiring targeted treatment. Quantitatively, our review indicates that these errors are non-negligible, potentially causing a horizontal wind speed underestimation of 0.4–3.4% and a turbulence intensity inflation of 15–50%. Second, the compensation toolbox has matured considerably over three decades, evolving from straightforward vector arithmetic to sophisticated sensor-fusion algorithms, hydrodynamic response models, and, most recently, machine-learning frameworks capable of learning error patterns that resist analytical description. Third, despite this progress, a significant gap remains between laboratory or calm-sea validation and robust, all-weather performance. Extreme conditions—precisely when observations are most valuable—continue to expose the limits of current hardware and algorithms.

Looking ahead, we see several priorities. On the algorithmic front, physics-informed neural networks offer a promising path toward models that are both flexible and interpretable; combining data-driven learning with embedded physical constraints may reduce the training-data burden while improving generalization. On the hardware side, lower-power inertial sensors, multi-antenna GNSS receivers, and actively stabilized platform designs could shrink error sources at the measurement stage rather than relying solely on post hoc correction. Perhaps most urgent is the need for community-level coordination: shared benchmark datasets, transparent validation protocols, and cross-platform intercomparison campaigns would accelerate progress far more than isolated, proprietary developments.

In sum, the challenges are substantial but not insurmountable. Continued collaboration between oceanographers, wind-energy engineers, sensor developers, and data scientists should enable buoy-based wind measurements to achieve the accuracy and reliability that both scientific research and commercial applications demand.

## Figures and Tables

**Table 1:** Characteristics of buoy-mounted wind measurement technologies and their intrinsic sensitivity to platform motion.

Anemometer Type	Measurement Principle	Key Advantages	Key Limitations	Intrinsic Sensitivity to Motion
Cup/Propeller Anemometer	Mechanical rotation proportional to wind speed	Simple structure, low cost, robust, widely deployed	Mechanical inertia, slow dynamic response, cannot resolve vertical wind components	High. Rotational speed is directly affected by tilt, acceleration, and relative wind components [32,33].
Ultrasonic Anemometer	Acoustic time-of-flight between transducers	Fast response, no moving parts, measures 3D wind vector	Flow distortion (shadowing) is sensitive to precipitation, higher power, and cost	Moderate. Sensitive to coordinate rotation and local flow distortion caused by platform motion [42].
Doppler Wind LiDAR	Doppler shift in backscattered laser beams	Remote sensing, elevated wind profiles, and avoiding local flow distortion	High cost and power consumption, sensitive to aerosols and precipitation	Extremely high. Platform motion induces beam misalignment and line-of-sight velocity errors [5,17,24,48].

**Table 2:** Comparative performance of attitude-error compensation methods.

Evaluation Dimension	Physical-Model Compensation	Multi-Sensor Data Fusion	RAO-Based Wave-Response Modeling	AI/ML-Based Compensation
Primary Objective	Correct orientation-induced geometric errors and relative-wind contamination	Provide robust, drift-resistant motion estimates (attitude/velocity) that enable physical-model-based kinematic correction	Provide redundant or supplementary motion information	Identify and compensate for nonlinear, coupled error sources
Accuracy for Mean Wind Speed/Direction	Moderate (limited by sensor precision)	High (effectively mitigates drift)	Moderate (dependent on hydrodynamic-model fidelity)	High potential (dependent on data quality and diversity)
Accuracy for Turbulence Intensity (TI)	Low (motion-induced turbulence remains unresolved)	Moderate (improves motion input yet does not eliminate aliasing)	Low (primarily effective for low-frequency motion)	High (capable of learning and disentangling nonlinear motion–turbulence features)
Real-Time Capability	High (minimal computational burden)	Medium–High (moderate filtering cost)	Moderate (requires wave-spectrum processing and RAO-based prediction)	Low (training expensive; inference latency may constrain deployment)
Computational Complexity	Low	Medium	Medium	High
Dependence on Sensor Precision	High	Medium (fusion reduces sensitivity to any single sensor)	Low (serves as redundancy during sensor degradation or failure)	Medium (requires high-quality motion and wind-speed records for training)
Adaptability to Sea-State/Platform Variability	Low (fixed parameters)	Medium (requires retuning of filter parameters)	Medium (RAO must match platform design and sea-state conditions)	High (learns adaptable representations across varying conditions)
Interpretability	High (physically transparent)	Medium (filtering steps interpretable)	Medium (hydrodynamic basis clearly defined)	Low (intrinsically black-box)
Overall Assessment	Provides strong real-time performance but limited accuracy; forms the foundational layer for higher-level compensation methods	A critical enabling layer that improves the reliability of motion inputs for physical-model-based correction, thereby enhancing overall compensation performance	Enhances system robustness and is particularly valuable in operational floating measurement systems, including commercial FLS deployments, as a redundancy mechanism	Represents the most promising direction for addressing nonlinear, coupled error mechanisms—especially motion-induced turbulence—though dependent on extensive high-quality training datasets and challenged by limited interpretability

## Data Availability

No new data were created or analyzed in this study.

## References

[1] Jishad M., Ratheesh S., Agarwal N., Sharma N., Sharma R. (2025). Impact of spatial and temporal resolution of satellite sea surface salinity measurements on ocean state prediction in the Tropical Indian Ocean; an OSSE framework using SMOS. Adv. Space Res..

[2] Zang T., Zou J., Li Y., Qiu Z., Wang B., Cui C., Li Z., Hu T., Guo Y. (2024). Development and Evaluation of a Short-Term Ensemble Forecasting Model on Sea Surface Wind and Waves across the Bohai and Yellow Sea. Atmosphere.

[3] Babanin A.V. (2023). Ocean waves in large-scale air-sea weather and climate systems. J. Geophys. Res. Ocean..

[4] Wu L., Sahlée E., Nilsson E., Rutgersson A. (2024). A review of surface swell waves and their role in air–sea interactions. Ocean Model..

[5] Salcedo-Bosch A., Farré-Guarné J., Araújo da Silva M.P., Rocadenbosch F. (2023). A unified formulation for the computation of the six-degrees-of-freedom-motion-induced errors in floating doppler wind lidars. Remote Sens..

[6] Wen Y., Wu J., Lin P., Low Y.M. (2025). The role of offshore wind and solar PV resources in global low-carbon transition. Sci. Adv..

[7] Su X., Wang X., Xu W., Yuan L., Xiong C., Chen J. (2024). Offshore wind power: Progress of the edge tool, which can promote sustainable energy development. Sustainability.

[8] Pelser T., Weinand J.M., Kuckertz P., McKenna R., Linssen J., Stolten D. (2024). Reviewing accuracy & reproducibility of large-scale wind resource assessments. Adv. Appl. Energy.

[9] Thiébaut M., Thebault N., Le Boulluec M., Damblans G., Maisondieu C., Benzo C., Guinot F. (2024). Experimental evaluation of the motion-induced effects for turbulent fluctuations measurement on floating LiDAR systems. Remote Sens..

[10] McPhaden M.J., Connell K.J., Foltz G.R., Perez R.C., Grissom K. (2023). Tropical ocean observations for weather and climate. Oceanography.

[11] Liu Y., Ning C., Zhang Q., Yuan G., Li C. (2024). Research on ocean buoy attitude prediction model based on multi-dimensional feature fusion. Front. Mar. Sci..

[12] Li Y., Zhao C., Jing P., Chen B., He G., Zhang Z., Zhang J., Li M., Wang J. (2025). Estimation of the Motion Response of a Large Ocean Buoy in the South China Sea. J. Mar. Sci. Eng..

[13] Li Y., Yang F., Li S., Tang X., Sun X., Qi S., Gao Z. (2023). Influence of Six-Degree-of-Freedom Motion of a Large Marine Data Buoy on Wind Speed Monitoring Accuracy. J. Mar. Sci. Eng..

[14] Krishnamurthy R., García Medina G., Gaudet B., Gustafson W.I., Kassianov E.I., Liu J., Newsom R.K., Sheridan L.M., Mahon A.M. (2023). Year-long buoy-based observations of the air–sea transition zone off the US west coast. Earth Syst. Sci. Data Discuss..

[15] Collins C., Jensen R. (2022). Tilt error in NDBC ocean wave height records. J. Atmos. Ocean. Technol..

[16] Tucker M.J., Pitt E.G. (2001). Waves in Ocean Engineering.

[17] Kelberlau F., Mann J. (2022). Quantification of motion-induced measurement error on floating lidar systems. Atmos. Meas. Tech. Discuss..

[18] Wang D. (1997). Analysis of wind measurements from a moored buoy. Oceans’ 97. MTS/IEEE Conference Proceedings.

[19] Zhou Y., Wang G., Zhao Y. (2014). Research on error analysis and compensation of wind speed measurement for ships under swaying motions. Chin. J. Sci. Instrum..

[20] Huang Y., Song J., Fan C. (2013). Amotion correction on direct estimations of air-sea fluxes froma buoy. Acta Oceanol. Sin..

[21] Pond S. (1968). Some effects of buoy motion on measurements of wind speed and stress. J. Geophys. Res..

[22] Uchiyama S., Ohsawa T., Asou H., Konagaya M., Misaki T., Araki R., Hamada K. (2025). Empirical Motion Compensation for Turbulence Intensity Measurement by Floating LiDARs. Energies.

[23] Rapisardi G., Da Silva M.P.A., Miquel A. (2024). A Machine Learning Approach to Correct Turbulence Intensity measured by Floating Lidars. J. Phys. Conf. Ser..

[24] Malekmohammadi S., Duscha C., Jenkins A.D., Kelberlau F., Gottschall J., Reuder J. (2024). Evaluating the performance of pulsed and continuous-wave lidar wind profilers with a controlled motion experiment. Remote Sens..

[25] Barber S., Schubiger A., Koller S., Eggli D., Radi A., Rumpf A., Knaus H. (2022). The wide range of factors contributing to wind resource assessment accuracy in complex terrain. Wind. Energy Sci. Discuss..

[26] Gottschall J., Gribben B., Stein D., Würth I. (2017). Floating lidar as an advanced offshore wind speed measurement technique: Current technology status and gap analysis in regard to full maturity. Wiley Interdiscip. Rev. Energy Environ..

[27] Russell A.J., McMorland J., Collu M., McDonald A.S., Thies P.R., Keane A., Quayle A.R., McMillan D., Carroll J., Coraddu A. (2024). The Impact of LIDAR-Assisted Pitch Control on Floating Offshore Wind Operational Expenditure. Wind. Energy.

[28] Gräfe M., Pettas V., Dimitrov N., Cheng P.W. (2024). Machine-learning-based virtual load sensors for mooring lines using simulated motion and lidar measurements. Wind. Energy Sci..

[29] Turton J., Pethica C. (2010). Assessment of a new anemometry system for the met office’s moored buoy network. J. Atmos. Ocean. Technol..

[30] Mangano S., Vega E., Martínez A., Alfonso-Corcuera D., Sanz-Andrés Á., Pindado S. (2022). Performance Monitoring of Mast-Mounted Cup Anemometers Multivariate Analysis with ROOT. Sensors.

[31] Thomas B., Swail V. (2011). Buoy wind inhomogeneities related to averaging method and anemometer type: Application to long time series. Int. J. Climatol..

[32] Pedersen T.F., Dahlberg J.-Å. (2024). Modelling of cup anemometry and dynamic overspeeding in average wind speed measurements. Atmos. Meas. Tech..

[33] Sanuki M., Kimura S. (1955). Experiments on a Capp-Anemometer in Pitching and Rolling Motion. Pap. Meteorol. Geophys..

[34] Sanuki M., Kimura S. (1954). Experiments on a Marine Combination Wind Vane and Anemometer in Pitching or Rolling Motion. Pap. Meteorol. Geophys..

[35] Watson W., Wolken-Möhlmann G., Gottschall J. (2025). Evaluating the Impact of Motion Compensation on Turbulence Intensity Measurements from Continuous-Wave and Pulsed Floating Lidars. Wind. Energy Sci. Discuss..

[36] Brown C.W., Schadee M., de Haij M., Brandsma T. (2025). Performance and longevity of compact all-in-one weather stations–the good, the bad and the ugly. EGUsphere.

[37] Shan Z., Xie X., Liu X. (2023). Wind speed and direction measurement based on three mutually transmitting ultrasonic sensors. IEEE Geosci. Remote Sens. Lett..

[38] Wróblewski A., Banasiewicz A., Krot P., Trybała P., Zimroz R., Zinchenko A. (2025). A New Method of Airflow Velocity Measurement by UAV Flight Parameters Analysis for Underground Mine Ventilation. Sensors.

[39] Compere M.D., Adkins K.A., Muthu Krishnan A. (2023). Go with the flow: Estimating wind using uncrewed aircraft. Drones.

[40] Nosov V., Lukin V., Nosov E., Torgaev A., Bogushevich A. (2019). Measurement of atmospheric turbulence characteristics by the ultrasonic anemometers and the calibration processes. Atmosphere.

[41] Glabeke G., Gigon A., De Mulder T., Van Beeck J. (2024). How accurate are ultrasonic anemometers, calibrated in a laminar wind tunnel, under turbulent conditions?. J. Phys. Conf. Ser..

[42] Li H., Lin M., Liu Y., Ding Y., Chen W., Wang T., Lu J., Li X. (2025). Investigation of Shadow Effects in Reflective Ultrasonic Anemometers Based on Particle Image Velocimetry and Computational Fluid Dynamics. J. Appl. Fluid Mech..

[43] Sheridan L.M., Krishnamurthy R., Gustafson W.I., Liu Y., Gaudet B.J., Bodini N., Newsom R.K., Pekour M. (2024). Offshore low-level jet observations and model representation using lidar buoy data off the California coast. Wind. Energy Sci. Discuss..

[44] Zhao S., Shan Y. (2024). Research on attitude correction algorithm for mobile wind lidars. Meas. Sci. Technol..

[45] Yuan Y., He Y., Zhu B., Gu C., Song L., Chen D., Qiu J., Yang C. (2025). Analysis and Validation of Measurement Data from Floating Wind Measurement Systems Based on Continuous-Wave LiDAR. J. Appl. Oceanogr..

[46] Baidar S., Wagner T.J., Turner D.D., Brewer W.A. (2023). Using optimal estimation to retrieve winds from velocity-azimuth display (VAD) scans by a Doppler lidar. Atmos. Meas. Tech..

[47] Angelou N., Mann J., Dubreuil-Boisclair C. (2023). Revealing inflow and wake conditions of a 6MW floating turbine. Wind. Energy Sci. Discuss..

[48] Gräfe M., Pettas V., Gottschall J., Cheng P.W. (2023). Quantification and correction of motion influence for nacelle-based lidar systems on floating wind turbines. Wind. Energy Sci..

[49] Kim I.I., McArthur B., Korevaar E.J. (2001). Comparison of laser beam propagation at 785 nm and 1550 nm in fog and haze for optical wireless communications. Optical Wireless Communications III.

[50] Petraitis D.C., DiNapoli S.M. (2018). Comparison of the NDBC 2.1-meter SCOOP buoy to the operational 3-meter buoy. OCEANS 2018 MTS/IEEE Charleston.

[51] Wang J., Li Y. (2019). Development and Application of Marine Data Buoy Technology in China. Shandong Sci..

[52] Jiang C., el Moctar O., Schellin T.E. (2021). Mooring-configurations induced decay motions of a buoy. J. Mar. Sci. Eng..

[53] Cao Y., Wang K., Xi C., Wang F. (2024). Hydrodynamic performance characteristics of small-scale in-situ buoys with critical motion requirements. Ocean Eng..

[54] Jia W.G., Ji J.H., Zhang C., Chen F.F., Cheng S.H., Gao Z.K., Shen F.F., Yuan L.L. (2024). Calibration of satellite typhoon data based on attitude modified buoy. Terr. Atmos. Ocean. Sci..

[55] Centurioni L.R., Turton J., Lumpkin R., Braasch L., Brassington G., Chao Y., Charpentier E., Chen Z., Corlett G., Dohan K. (2019). Global in situ observations of essential climate and ocean variables at the air–sea interface. Front. Mar. Sci..

[56] Zippel S., Thomson J. (2017). Surface wave breaking over sheared currents: Observations from the Mouth of the Columbia River. J. Geophys. Res. Ocean..

[57] Nassif F.B., Pimenta F.M., Assireu A.T., D’Aquino C.D.A., Passos J.C. (2020). Wind measurements using a LIDAR on a buoy. RBRH.

[58] Viselli A., Filippelli M., Pettigrew N., Dagher H., Faessler N. (2019). Validation of the first LiDAR wind resource assessment buoy system offshore the Northeast United States. Wind Energy.

[59] Yu T., Li T., Shi H., Zhang Z., Chen X. (2023). Experimental investigation on the wave-oscillating buoy interaction and wave run-up on the buoy. Ocean Eng..

[60] Liu T., Xiang M., Zhou B., Zhang X. (2025). Hydrodynamic performance of a multi-cylinder LiDAR buoy prototype for wind assessment. J. Ocean Eng. Mar. Energy.

[61] Li X., Bian Y. (2021). Modeling and prediction for the Buoy motion characteristics. Ocean Eng..

[62] Rainville E., Thomson J., Moulton M., Derakhti M. (2023). Measurements of nearshore ocean-surface kinematics through coherent arrays of free-drifting buoys. Earth Syst. Sci. Data.

[63] Villarreal-Olavarrieta C.E., Ocampo-Torres F.J., Osuna P., Mora-Escalante R.E. (2024). Effect of waves on the magnitude and direction of wind stress over the Ocean. Ocean Model..

[64] Li Y., Zhou J., Wang H., Wang C. (2025). Influence of Viscous Effects on Mooring Buoy Motion. J. Mar. Sci. Eng..

[65] Riihimaki L.D., Cronin M.F., Acharya R., Anderson N., Augustine J.A., Balmes K.A., Berk P., Bozzano R., Bucholtz A., Connell K.J. (2024). Ocean surface radiation measurement best practices. Front. Mar. Sci..

[66] Faltinsen O. (1993). Sea Loads on Ships and Offshore Structures.

[67] Hamilton G.D. (1980). NOM Data Buoy Office Programs. Bull. Am. Meteorol. Soc..

[68] Edson J.B., Hinton A.A., Prada K.E., Hare J.E., Fairall C.W. (1998). Direct covariance flux estimates from mobile platforms at sea. J. Atmos. Ocean. Technol..

[69] Lumpkin R., Pazos M. (2007). Measuring surface currents with Surface Velocity Program drifters: The instrument, its data, and some recent results. Lagrangian Anal. Predict. Coast. Ocean Dyn..

[70] Wright E.E., Bourassa M.A., Stoffelen A., Bidlot J.-R. (2021). Characterizing buoy wind speed error in high winds and varying sea state with ASCAT and ERA5. Remote Sens..

[71] Wilczak J.M., Oncley S.P., Stage S.A. (2001). Sonic anemometer tilt correction algorithms. Bound.-Layer Meteorol..

[72] Miller S.D., Hristov T.S., Edson J.B., Friehe C.A. (2008). Platform motion effects on measurements of turbulence and air–sea exchange over the open ocean. J. Atmos. Ocean. Technol..

[73] Wolken-Möhlmann G., Bischoff O., Gottschall J. (2022). Analysis of wind speed deviations between floating lidars, fixed lidar and cup anemometry based on experimental data. J. Phys. Conf. Ser..

[74] Smith S.R., Bourassa M.A., Sharp R.J. (1999). Establishing more truth in true winds. J. Atmos. Ocean. Technol..

[75] Kelberlau F., Neshaug V., Lønseth L., Bracchi T., Mann J. (2020). Taking the motion out of floating lidar: Turbulence intensity estimates with a continuous-wave wind lidar. Remote Sens..

[76] Benzo C., Delbos F., Yahiaoui S. (2024). Applying motion compensation to offshore wind lidar reconstructed wind measurements. J. Phys. Conf. Ser..

[77] Li Z., Wang N., Qi S., Zhang Z., Sun J., Wang D. (2020). Research on correction method of wind measurement based on platform attitude. J. Phys. Conf. Ser..

[78] Yurovsky Y.Y., Dulov V.A. (2020). MEMS-based wave buoy: Towards short wind-wave sensing. Ocean Eng..

[79] Farahan S.B., Machado J.J., de Almeida F.G., Tavares J.M.R. (2022). 9-DOF IMU-based attitude and heading estimation using an extended Kalman filter with bias consideration. Sensors.

[80] Spiliotis E., Theodorou E. (2025). Improving wind power forecasting accuracy through bias correction of wind speed predictions. Sustain. Energy Technol. Assess..

[81] Flügge M., Paskyabi M.B., Reuder J., Edson J.B., Plueddemann A.J. (2016). Comparison of direct covariance flux measurements from an offshore tower and a buoy. J. Atmos. Ocean. Technol..

[82] Salcedo-Bosch A., Rocadenbosch F., Sospedra J. (2021). A robust adaptive unscented kalman filter for floating doppler wind-lidar motion correction. Remote Sens..

[83] Jouybari A., Ardalan A., Rezvani M.-H. (2017). Experimental comparison between Mahoney and Complementary sensor fusion algorithm for attitude determination by raw sensor data of Xsens IMU on buoy. Int. Arch. Photogramm. Remote Sens. Spat. Inf. Sci..

[84] Libero Y., Klein I. (2025). Attitude and Heading Estimation in Symmetrical Inertial Arrays. arXiv.

[85] Yu W., Bischoff O., Cheng P.W., Wolken-Moehlmann G., Gottschall J. (2018). Validation of a Simplified LiDAR-Buoy Model Using Open Sea Measurements. International Conference on Offshore Mechanics and Arctic Engineering.

[86] Mathias N., Marini R.N., Morais T., Luís P., Vaz M., Rosa-Santos P. (2021). Response of a self-powered offshore floating support structure with an OWC for powering a LIDAR device. Ocean Eng..

[87] Yu J., Han T., Li L., Shao Y. (2017). Current Status of Hydrodynamic Characterization of Ocean Data Buoys. Trans. Oceanol. Limnol..

[88] Brown A.C., Paasch R.K. (2021). The accelerations of a wave measurement buoy impacted by breaking waves in the surf zone. J. Mar. Sci. Eng..

[89] Campos R.M., Islam H., Ferreira T.R., Soares C.G. (2021). Impact of heavy biofouling on a nearshore heave-pitch-roll wave buoy performance. Appl. Ocean Res..

[90] Islam H., Campos R.M., Ferreira T.R., Guedes Soares C. (2020). Hydrodynamic assessment of a biofouled wave buoy in coastal zone. International Conference on Offshore Mechanics and Arctic Engineering.

[91] Yin Y., Zhang J., Guo M., Ning X., Wang Y., Lu J. (2023). Sensor fusion of GNSS and IMU data for robust localization via smoothed error state Kalman filter. Sensors.

[92] Song J., Li X., Zhang Z., Zhao Z., Wang C., Wu S., Qi S., Lv J. (2024). A predictive framework for shipborne wind speed measurement correction based on self-supervised contrastive learning. 2024 27th International Conference on Computer Supported Cooperative Work in Design (CSCWD).

[93] Wang J., Wang Z., Wang Y., Liu S., Li Y. (2016). Current situation and trend of marine data buoy and monitoring network technology of China. Acta Oceanol. Sin..

[94] Azorin-Molina C., Asin J., McVicar T.R., Minola L., Lopez-Moreno J.I., Vicente-Serrano S.M., Chen D. (2018). Evaluating anemometer drift: A statistical approach to correct biases in wind speed measurement. Atmos. Res..

[95] Zhang Y., Kong X., Wang J., Wang S., Zhao Z., Wang F. (2024). A comprehensive wind speed prediction system based on intelligent optimized deep neural network and error analysis. Eng. Appl. Artif. Intell..

